# Genetic Findings as the Potential Basis of Personalized Pharmacotherapy in Phelan-McDermid Syndrome

**DOI:** 10.3390/genes12081192

**Published:** 2021-07-30

**Authors:** Brianna Dyar, Erika Meaddough, Sara M. Sarasua, Curtis Rogers, Katy Phelan, Luigi Boccuto

**Affiliations:** 1 Healthcare Genetics Program, School of Nursing, Clemson University, Clemson, SC 29634, USA; bhdyar@g.clemson.edu (B.D.); emeaddo@g.clemson.edu (E.M.); smsaras@clemson.edu (S.M.S.); 2 Greenwood Genetic Center, Greenwood, SC 29649, USA; crogers@ggc.org; 3 Florida Cancer Specialists & Research Institute, Fort Myers, FL 33905, USA; kphelan@flcancer.com

**Keywords:** Phelan-McDermid syndrome, autism, therapy, *CYP2D6*, *SHANK3*

## Abstract

Phelan-McDermid syndrome (PMS) is a genetic disorder often characterized by autism or autistic-like behavior. Most cases are associated with haploinsufficiency of the *SHANK3* gene resulting from deletion of the gene at 22q13.3 or from a pathogenic variant in the gene. Treatment of PMS often targets *SHANK3*, yet deletion size varies from <50 kb to >9 Mb, potentially encompassing dozens of genes and disrupting regulatory elements altering gene expression, inferring the potential for multiple therapeutic targets. Repurposed drugs have been used in clinical trials investigating therapies for PMS: insulin-like growth factor 1 (IGF-1) for its effect on social and aberrant behaviors, intranasal insulin for improvements in cognitive and social ability, and lithium for reversing regression and stabilizing behavior. The pharmacogenomics of PMS is complicated by the CYP2D6 enzyme which metabolizes antidepressants and antipsychotics often used for treatment. The gene coding for CYP2D6 maps to 22q13.2 and is lost in individuals with deletions larger than 8 Mb. Because PMS has diverse neurological and medical symptoms, many concurrent medications may be prescribed, increasing the risk for adverse drug reactions. At present, there is no single best treatment for PMS. Approaches to therapy are necessarily complex and must target variable behavioral and physical symptoms of PMS.

## 1. Introduction

Phelan-McDermid syndrome (PMS) is a neurodevelopmental disorder frequently associated with autism spectrum disorder (ASD), seizures, and variable dysmorphic features. It is typically caused by deletions of 22q13.3. Pathogenic variants of a gene contained within this region, *SHANK3*, have also been reported. *SHANK3* encodes a scaffolding protein that is enriched in postsynaptic densities of excitatory neurons [1].

Young children typically present with delayed motor milestones, hypotonia, dysplastic toenails, and absent to severely delayed speech. As they get older, they may develop large fleshy hands, long eyelashes, and decreased perspiration that results in a tendency to overheat. Behavioral characteristics include mouthing or chewing non-food items, decreased perception of pain, and autism or autistic-like affect and behavior. Most affected individuals have moderate to profound intellectual disability. Developmental and intellectual regression has been reported in several individuals, usually beginning in adolescence or adulthood. Seizures, sleep disturbances, lymphedema, and gastrointestinal issues can also develop later in life and may result in significant health problems [1,2,3,4,5].

Autism spectrum disorder is a major neurobehavioral component of PMS and may be present in 75–80% of individuals with this condition [1]. As expected, the significant intellectual disability seen in PMS contributes to the neurobehavioral phenotype. Pathogenic variants in the synaptic scaffolding protein gene *SHANK3* are strongly implicated in autism and Phelan–McDermid 22q13 deletion syndrome and have been associated with abnormal glutamate receptor function and spine morphology. SHANK3 also interacts with the Neurexin-Neuroligin signaling complex in hippocampal neurons and plays a role in regulating AMPA and NMDA receptor-mediated excitatory synaptic transmission [6,7]. Wang et al. demonstrated in mice that even missense variants in *SHANK3* may play a role in ASD and that *SHANK3* functions in several independent pathways which may make treatment difficult [8].

The prevalence of PMS is unknown. However, it appears to be one of the more common chromosome deletion syndromes and well over 3000 individuals have been reported worldwide. The clinical variability of the physical and intellectual features may allow individuals to go undiagnosed, but the advent of chromosomal microarrays and exome sequencing has dramatically increased the detection rate.

The wide clinical spectrum has been ascribed, at least in part, to the extreme variability in the size of the 22q13.3 deletion which may range from less than 50 kb to greater than 9 Mb [1,2,3,4,9,10,11,12,13]. Obviously, these deletions contain a variable number of genes that may potentially impact both the phenotype and response to therapies.

## 2. Pathogenic Molecular Mechanisms in PMS

Since most cases of PMS have been associated with terminal 22q13.3 deletions or pathogenic variants in the *SHANK3* gene, haploinsufficiency of this gene is considered the main cause of the major neurodevelopmental features of the syndrome, such as hypotonia, seizures, or behavioral issues [1,2,3,12]. Due to its role in the postsynaptic density of excitatory synapses, *SHANK3* has been considered an ideal target for treatment, leading to approaches aiming to increase the expression of the wild-type allele or stabilize/strengthen the synapses. However, variants in *SHANK3* have been associated with a wide spectrum of conditions in addition to PMS, from isolated autism spectrum disorder to schizophrenia, from non-syndromic intellectual disability to a Rett-like phenotype or Alzheimer’s disease [14,15,16,17,18,19]. Moreover, it has been proven that not all pathogenic variants of *SHANK3* cause a loss of function. Indeed, some variants may even lead to a gain-of-function effect [20], suggesting an imbalance in the interaction of SHANK3 with other proteins of the postsynaptic density that does not depend on haploinsufficiency. Finally, most individuals (approximately 75%) with PMS carry a 22q13.3 deletion greater than 1 Mb [3,9], causing the loss of dozens of genes, in addition to *SHANK3*. The loss of one copy of these genes is expected to contribute to the clinical presentation of the syndrome, as suggested by genotype-phenotype analyses indicating a positive correlation between the size of the deletion and the number and/or severity of certain clinical traits, such as developmental delay [10,11,12], autistic traits [3], speech/language abilities [4], hypotonia [10,11,12], and dysmorphic features [3,12]. In some cases, interstitial 22q13.3 deletions, not encompassing *SHANK3*, have been described in association with a clinical presentation overlapping with some PMS phenotypes [21,22,23]; this suggests the presence of certain genes, proximal to *SHANK3*, whose haploinsufficiency can cause similar neurobehavioral issues as those observed in the cases with terminal deletions or *SHANK3* pathogenic variants. The potential additive or contributive role of these genes must be considered in the pathogenic mechanisms of cases with large deletions. 

Chromosomal rearrangements other than the simple terminal deletions are responsible for some cases of PMS, calling for specific protocols of clinical management and treatment. For example, it has been estimated that about 14% of individuals with this syndrome carry a ring 22 chromosome (r22) and a recent study reports that 16% of subjects with r22 either received a diagnosis of Neurofibromatosis 2 (NF2) or had an NF2-associated tumor [24]. Clinical surveillance and therapeutic approaches in cases with r22 should therefore consider the risk of NF2 in addition to the typical features of PMS.

22q13.3 deletions larger than 1 MB may also disrupt regulatory elements and lead to altered expression of genes either within the same region or in other genomic loci, through dysregulated methylation [25]. Data from the PMS Foundation International Registry indicate that additional copy number variants (CNVs) are present in approximately 25% of individuals, which may contribute to the variability of the clinical presentation of the syndrome and complicate the development of a targeted treatment. 

Some studies have identified specific genes within the commonly deleted 22q13.3 region as potential contributors to the PMS phenotypes [26,27], suggesting, among the others, a role for the *SULT4A1* gene in speech delay and abnormal cerebellar development and function, *BRD1* in neuropsychiatric disorders [28], and *GRAMD4*, *SCO2*, and *TYMP* in mitochondrial abnormalities [29]. Moreover, the loss of one allele of the *PNPLA3* gene due to a large 22q13.3 deletion can unmask a heterozygous variant, increasing the risk of liver disease, leading to gastrointestinal issues and abnormal responses to various drugs [30].

In consideration of such variability in the pathogenic molecular mechanisms observed in PMS, therapeutic strategies aiming to address the consequences of *SHANK3* haploinsufficiency may be promising for some of the neurodevelopmental traits of individuals with PMS but may fail to solve several other problems, particularly in subjects with large 22q13.3 deletions. Personalized therapeutic approaches for patients with PMS must therefore consider the complicated genomic profile and the constellation of potential genetic and epigenetic factors contributing to the phenotype observed in each subject.

## 3. Challenges of Drug Development in PMS

Identifying potential therapies in PMS encounters many of the same challenges that confront drug development in other conditions. These include demonstrating the safety, efficacy and pharmacokinetics of the drug or agent proposed for treatment. However, in PMS and other neurodevelopmental disorders, the major hurdle is developing therapeutic agents that penetrate the central nervous system. Two barrier systems are present in the brain: the blood–brain barrier (BBB), formed by the brain microvasculature, and the blood–cerebrospinal fluid (CSF) barrier, formed by the choroid plexus epithelium [31]. Oral and intravenous drug administration allows for the measurement of drug concentration in the bloodstream. The drug may pass through the blood-CSF barrier, but this is not an indication that the drug will reach the brain. Small and large molecules in the blood enter the CSF at a rate inversely related to their molecular weight but may not cross the blood–brain barrier due to efflux transport actions at the brain microvasculature. Direct injection into the cerebrospinal is another potential route of drug delivery. While this method circumvents the blood-CSF barrier, the drug is rapidly exported to the blood with minimal delivery to the brain parenchyma which is mediated by diffusion, a slow process that decreases exponentially with distance [31]. 

Born et al. [32] demonstrated that intranasal administration of neuropeptides was an effective means of achieving biologically effective concentrations in the brain without strong systemic side effects. They administered three peptides—melanocortin, vasopressin, and insulin—intranasally and measured direct access to the CSF. The mean CSF concentration began to rise within 10 min of intranasal administration and peaked at 30 min for melanocortin and insulin and 80 min for vasopressin. Higher doses resulted in prolonged drug retention. The increased concentrations in the CSF did not correlate with an increased concentration in the bloodstream, suggesting that the peptides bypassed the bloodstream while entering the CSF. The authors suggest that the peptides pass from the nose to the brain by an extra-neuronal pathway. By this pathway, the molecules traverse the patent intercellular clefts in the olfactory epithelium to diffuse into the subarachnoid space. Furthermore, Born et al. [32] cite animal studies that have shown rapid accumulation of peptides in cerebral and spinal CSF and brain tissue within 10–20 min of administration, giving credence to the use of intranasal administration of drugs in clinical trials.

More recently, nanotechnology-based approaches have been proposed as a means of crossing the BBB. This methodology utilizes therapy-loaded nanoparticles to penetrate the BBB and deliver drugs to the brain for the treatment of neurological disorders. However, the administration of the therapeutic nanoparticles is problematic. Injection of nanoparticles into the bloodstream has met with limited success in animal studies, as the ligands associated with the nanoparticles also bind to targets on peripheral organs, reducing the amount of drug ultimately reaching the brain [33]. Research is now focusing on ways to enhance the permeability of the BBB and to selectively target the brain epithelium with these therapeutic particles [34].

## 4. Ongoing Strategies in Treatment of Neurobehavioral Symptoms in PMS

Currently, treatment for PMS is symptomatic using symptom-specific guidelines [35,36,37]. While no therapeutic protocol has been specifically developed for PMS so far, several repurposed medications are being investigated for use in PMS treatment, particularly growth hormones, intranasal insulin, lithium, and other FDA-approved medications.

### 4.1. Growth Hormones IGF-1 and rGH

Insulin-like growth factor 1 (IGF-1) is an FDA-approved treatment for Laron’s dwarfism; however, studies in mouse models imply that it may also increase synaptic plasticity and improve both motor and behavioral deficits characteristic of PMS and other autism spectrum disorders (ASD) [38]. The first clinical trial of IGF-1 as a treatment for PMS was carried out by Dr. Alexander Kolevzon and colleagues at the Seaver Autism Center for Research and Treatment. They recruited nine children aged 5–15 with confirmed haploinsufficiency of the *SHANK3* gene (deletions or mutations) and subcutaneously administered a maximum dose of 0.12 mg/kg twice daily over three months in a placebo-controlled, double-blind, crossover design study. Post-treatment analysis revealed limited adverse effects and a significant improvement in both the social and aberrant behaviors of the patients as measured by the Aberrant Behavior Checklist (ABC) and the Repetitive Behavior Scale (RBS), respectively [39]. A follow-up phase II study on IGF-1 derived from subcutaneously injected recombinant human growth hormone rGH concluded in June 2020 and is currently pending results. This follow-up clinical trial examined patients using parameters outlined in the ABC and RBS again as well as an additional two: The Sensory Profile (TSP) and The Sensory Assessment for Neurodevelopmental Disorders (SAND) [40]. Additionally, a report published in 2021 describes the case study of a twenty-one-month-old Chinese PMS patient with a 34 kb deletion encompassing *SHANK3* prescribed rGH at a dosage of 0.075 IU/kg daily via subcutaneous injection for three months. At the conclusion of the treatment, her serum IGF-1, IGF-1-binding protein, and development quotient (DQ) all improved significantly, and more socially appropriate behavior was observed by her caretakers [41].

### 4.2. Intranasal Insulin

Intranasal insulin was tried in children with insulin-dependent diabetes mellitus and has recently been discovered to improve the cognitive status of patients with Alzheimer’s disease; The pilot trial for the treatment of PMS with intranasal insulin was conducted in 2008 by Schmidt et al. [42] where over the course of one year six children with 22q13.3 deletions were administered a dose of 0.5–1.5 IU/kg of intranasal insulin per day. Parents were asked to fill out a questionnaire at both the six-week and twelve-month milestones; improvements in the children’s motor and cognitive development were noted [42]. Building upon these findings, Zwanenburg and colleagues performed a larger, double-blind, placebo-controlled trial in 2016 that included twenty-five individuals aged one to sixteen with 22q13.3 deletions including *SHANK3*. In a step-wedge fashion, the children were administered either a placebo or 20–40 IU per day of insulin over a sixth month period, after which the rate of improvement was determined using standardized surveys; results showed a significant increase in developmental rates for cognitive and social ability in children aged three and over [43]. Since the intranasal method of administration bypasses the BBB, only minor side effects were noted in these studies such as the occasional nosebleed or headache [42,43]. 

### 4.3. Lithium

There are a few reports of the successful use of lithium in PMS. Darville et al. [44] used human pluripotent stem cell-derived cortical neurons to screen multiple compounds for the potential to increase *SHANK3* mRNA content. Lithium and valproic acid had the best efficacy in vitro. Treatment of a patient with lithium for one year showed decreased autism severity [44]. Other reports include case studies of individual patients (treatment of a 70-year-old woman with a 610 kb deletion [45] and a 13-year-old girl with a *SHANK3* microdeletion [46]. A recent case series for the treatment of PMS with lithium was reported in 2015 by researchers Serret and colleagues [47]. Patients were aged twenty-one and seventeen with *SHANK3* microdeletion and point mutation, respectively; they had suffered catatonic regression for which both had been previously treated with other drugs including benzodiazepines, antidepressants, and antipsychotics. The twenty-one-year-old patient was administered 1500 mg per day over the course of two years with the addition of 4 mg per day of melatonin for the first three months. By the end of the two years, his developmental level was restored to that which it was prior to regression. The seventeen-year-old patient was administered 1000 mg per day of lithium for the course of one year with an accompanying 0.2 mg per day dose of clonazepam for the first four months; her regression was reversed, and her behavior afterward remained stable. Assessments for patients both pre- and post-treatment were carried out using the Vineland Adaptive Behavior Scale (VABS) and the Clinical Global Impression Scale [47]. A similar study conducted by Egger et al. in 2017 describes the treatment of a forty-year-old PMS patient with a *SHANK3* point mutation experiencing regression with 700 mg of lithium and 10 mg of olanzapine a day; after one year, mood, behavior, and affect stabilized in the patient [48]. Two case studies were conducted in 2018 by Rowland et al. [49] The first patient was a fifty-four-year-old PMS patient with a 145 kb deletion including *SHANK3* and chronic kidney disease. Due to the comorbidity, lithium administration was gradually increased from 400 mg per day to 600 mg. Several weeks of observation revealed no kidney impairment related to the lithium medication and the patient exhibited less aggressive behavior. The other patient included in this study was aged seventeen with a 31 kb deletion including a portion of *SHANK3* and began demonstrating hyperactive behavior and dysregulated sleeping patterns. He was administered a subtherapeutic dose of lithium of 100 mg per day for a week with an increase to 400 mg per day throughout his time at an inpatient facility. Despite the low dosage, his previous symptoms were diminished and the patient showed significant improvement in behavior [49]. Following the success of these studies, a clinical trial is currently being organized as of 2021 by Delorme for lithium in the treatment of PMS [50].

### 4.4. Other Drugs

There are other pharmaceuticals under investigation as a treatment for PMS that have reached the human trial stage of testing, although the studies for these drugs may not be specifically devoted to PMS research prior to clinical trials. Ubiquinone (also referred to as CoQ10) has been used to treat patients with generalized autism spectrum disorder on the premise that it may help reduce oxidative stress in the brain and ameliorate ASD symptoms [51]. Following up on these promising studies, Dr. Persico and the Associazione Italiana Sindrome di Phelan-McDermid (AISPHEM) have begun recruiting PMS patients for a CoQ10 clinical trial [52]. Other drugs undergoing clinical trials for PMS include AMO-01 [53] and intranasal oxytocin [54].

## 5. Pharmacogenomics in PMS with Particular Focus on *CYP2D6* Located at 22q13.2

The field of pharmacogenomics focuses on the identification of genetic variants which impact drug metabolism and patient response to drug therapy. Implementation of pharmacogenetic testing in clinical management can be used as a tool in personalized care, with the potential to improve health outcomes and avoid adverse drug reactions. As mentioned previously there are no established therapies for PMS, therefore symptoms are treated individually. Because there is a wide range of conditions associated with PMS, such as depression, epilepsy, bipolar disorder, gastrointestinal disorders, and others, patients may be prescribed multiple concurrent medications, also known as polypharmacy. Polypharmacy is associated with an increased risk for adverse drug reactions [55].

Since *SHANK3* mutations have been associated with neuropsychiatric disorders more prevalent than PMS [56,57] it is fortunate that the role of pharmacogenetic considerations in treatment has been described in detail [58,59,60]. For instance, patients prescribed the anticonvulsant carbamazepine who test positive for variants of the HLA-A or HLA-B genes (specifically HLA-A*31:01 or HLA-B*15:02) are at risk for serious adverse reactions [61]. The Food and Drug Administration (FDA) includes pharmacogenetic information on the product labels for antipsychotics such as aripiprazole [62], which may be used to treat patients with schizophrenia [58,59]. Experts in the pharmacogenomics field also note that several other factors may modify the impact of genetic variants on drug efficacy. These include age, sex, disease status, and the use of multiple medications, among others [60,63]. Therefore, pharmacogenetic testing only provides an additional tool in an effort to improve therapeutic outcomes.

Cytochrome p450 (CYP) enzymes are particularly important for pharmacogenomic effects. These proteins play an significant role in the biotransformation of drugs and xenobiotics. They are membrane-bound hemeproteins found in all tissues but mainly expressed in the liver and small intestines [64]. While there are 18 known families of CYP enzymes in humans, CYP families 1, 2, and 3 are most active in drug metabolism. The FDA, the Clinical Pharmacogenetics Implementation Consortium (CPIC) and other professional groups recognize genes for CYP enzymes such as *CYP2C9*, *CYP2C19*, *CYP2D6*, *CYP3A4*, and *CYP3A5* as important pharmacogenes. *CYP2D6* is of particular importance. There are over 100 known variants of *CYP2D6* (e.g., indels, SNPs, CNVs), which metabolizes 20% of approved drugs, including several prescribed for psychiatric conditions [65]. Further, the *CYP2D6* gene is located in the 22q13.2 (22:42,126,498-42,130,809 hg38) region, which can have implications for PMS patients with large deletions or interstitial deletions in 22q13.2. Although there are many factors that contribute to drug response, established genotype-phenotype relationships allow for patients to be categorized into metabolizer groups based on *CYP2D6* variant status. Patient assignment to one of four groups, defined as ultra-rapid metabolizers, extensive (normal) metabolizers, intermediate metabolizers, and poor metabolizers, can alert clinicians as to whether dose adjustment or alternative drug therapies are warranted [65].

Medication management in patients with PMS can include the use of antidepressants, antipsychotics, and epileptics. Genetic variants of *CYP2D6* and other pharmacogenes, can have a significant impact on drug efficacy. In fact, pharmacogenetic guidelines for 17 neuropsychiatric related drugs are available through CPIC or the Dutch Pharmacogenetics Working Group based on *CYP2D6* variant status [65]. The poor metabolizer phenotype has been associated with differential clinical response compared to normal metabolizers (NMs). Clinicians are advised to prescribe an alternative to tricyclic antidepressants such as nortriptyline for patients who are poor metabolizers (PMs). Low metabolic activity can lead to potential side effects caused by higher plasma concentrations of the drug. Alternatively, the starting dose should be reduced to 50% of the normal dose, followed by dose adjustment after drug monitoring [66]. A reduction to half of the normal starting dose is also recommended for PMs taking aripiprazole, an antipsychotic drug [62] Additionally, the FDA recommends that the adult dose of pimozide, another antipsychotic, should not exceed 4 mg/day in PMs [67]. PMs prescribed atomoxetine for Attention Deficit Hyperactivity Disorder are also at risk for adverse reactions. In adults, Brown et al. [68] report a starting dose of 40 mg/day, followed by monitoring for two weeks before dose adjustment. An increase in dose from 40 mg/day to 80 mg/day is recommended for NMs only three days after initiation of treatment. Several public resources are available in recognition of the need to translate knowledge of pharmacogenetic variants to clinical practice [69,70]. Table 1 lists examples of medications that have been prescribed for PMS patients [37,71,72,73,74] as well as published clinical guidelines from CPIC. Pharmacogenetic testing for clinically relevant genetic variants could be useful in the clinical management of PMS patients [27].

ADHD, Attention Deficit Hyperactivity Disorder; *CYP2D6*, cytochrome P450 family 2 subfamily D member 6; *CYP2C9*, cytochrome P450 family 2 subfamily C member 9, *CYP2C19*, cytochrome P450 family 2 subfamily C member 19; HLA-A, major histocompatibility complex, class I, A; HLA-B, major histocompatibility complex, class I, B; UM, ultra-rapid metabolizer; PM, poor metabolizer; CPIC, Clinical Pharmacogenetics Implementation Consortium.

## 6. Discussion

The bulk of research into PMS phenotypes is primarily focused on neurobehavioral aberrations attributed to the loss of *SHANK3*. However, patients with interstitial deletions in chromosome 22 that do not include *SHANK3* still present with neurological symptoms typical of PMS. This suggests the presence of other genes on chromosome 22 that contribute to the stereotypical PMS phenotype, likely candidates being *SULT4A1* and *BRD1*. Additionally, individuals with PMS with 22q13 deletions larger than 6–8 Mb are also at risk of developing gastrointestinal problems since their deletion is large enough to include *PNPLA3*, and the loss of both *PNPLA3* and *CYP2D6* have been implicated in reducing a patient’s ability to metabolize various pharmaceuticals.

With regard to *CYP2D6*, genetic testing to infer metabolizer status may offer some assistance in the clinical management of patients with PMS. However, barriers to clinical implementation of pharmacogenetic testing have generally been difficult to overcome. Studies designed to uncover associations between genetic variants and therapeutic response can be limited by low patient sample size, possible sources of confounding such as population stratification, and the inability to replicate results [77]. In addition, pharmacogenomics expertise among clinicians is lacking, there can be inconsistent reporting of guidelines among professional organizations, and access to laboratories with genetic testing capabilities is not uniform [78]. Fortunately, the *CYP2D6* gene has been the subject of extensive research and is widely accepted as clinically significant in the pharmacogenomics community.

Apart from protein coding genes, the 22q13 region also contains regulatory elements responsible for methylation patterns for genes within 22q13, as well as other loci outside this region. The loss of these elements exerts epigenetic changes in the expression of an individual’s genome that may result in deleterious symptoms. While the traditional chromosomal model for PMS is a simple terminal deletion, loss of genetic material has also been observed in the form of a ring chromosome (r22), in which both the distal long arm and distal short arm are deleted. Due to the instability of ring chromosomes in cell division, the ring can be lost in somatic cells leading to monosomy for chromosome 22. A subsequent mutation at the *NF2* locus at 22q12 on the remaining copy of chromosome 22 can confer NF2 in addition to PMS.

## 7. Conlusions

After accounting for the mechanism of deletion and the size of the genetic loss, individuals with PMS occupy a broad spectrum of disease severity, yet the most common treatment approach is the reappropriation of existing pharmaceuticals that primarily treat the neurobehavioral symptoms attributed to the loss of *SHANK3*. The preference for this approach is most likely a consequence of their general safety and prior success in navigating the BBB and other obstacles to drug delivery. However, by focusing treatment efforts on one gene, many other potential targets, like those described above, are overlooked. Therefore, it is imperative that future research endeavors to stratify patients into categories based on an individual’s unique symptoms and the specific set of genes they are missing. By tackling diagnosis from a genomic angle, physicians will be able to puzzle together a more complete clinical picture for their patients.

## Figures and Tables

**Table 1:** Examples of medications prescribed in PMS and relevant pharmacogenetic guidelines.

Drug Class	Drug	Gene	Risk	CPIC Recommended Dosing Guidelines
Antidepressants	Nortriptyline Paroxetine Fluvoxamine Citalopram	*CYP2D6* *CYP2D6* *CYP2D6* *CYP2C19*	Therapeutic failure in UMs; side effects for PMs	[66] [75] [75] [75]
Anticonvulsants	Carbamazepine and Oxcarbazepine	*HLA-A* *HLA-B*	Maculopapular exanthema, drug reaction with eosinophilia and systemic systems, Stevens-Johnson syndrome, and toxic epidermal necrolysis in patients with HLA-A*31:01 or HLA-B*15:02 variants	[61]
	Phenytoin	*CYP2C9 HLA-B*	Stevens-Johnson syndrome and toxic epidermal necrolysis in patients with HLA-B*15:02 variant; toxicity in CYP2C9 poor metabolizers	[76]
ADHD	Atomoxetine	*CYP2D6*	Therapeutic failure in UMs; side effects for PMs	[68]

## Data Availability

No data were generated.
